# Complex Renal Cysts (Bosniak ≥ IIF): Outcomes in a Population-Based Cohort Study

**DOI:** 10.3390/cancers12092549

**Published:** 2020-09-08

**Authors:** James Lucocq, Sanjay Pillai, Richard Oparka, Ghulam Nabi

**Affiliations:** 1 Research Division of Imaging Sciences and Technology, School of Medicine, Ninewells Hospital and Medical School, Dundee DD19SY, UK; 2 Department of Radiology, Ninewells Hospital and Medical School, Dundee DD19SY, UK; sanjaypillai@nhs.net; 3 Department of Pathology, Ninewells Hospital and Medical School, Dundee DD19SY, UK; roparka@nhs.net; 4 Research Division of Imaging Sciences and Technology, Ninewells Hospital and Medical School, Dundee DD19SY, UK; gnabi@dundee.ac.uk

**Keywords:** kidney neoplasms, nephrectomy, survival analysis

## Abstract

**Simple Summary:**

Researchers from the University of Dundee have found that not all kidney cancers will need urgent surgery. In a published research in the Cancers, Dr Lucocq et al have carefully established database record of patients with kidney cancer looking like water filled sacs on CT scan and reported that these cancers are low grade and perhaps less harmful on long-term follow-up. In fact, behaviour of these cancer cells is much slower compared to other diseases such as heart failure and high blood pressure. Most people die from chronic disease much before cancer spread or progression. The researchers in this group have shown that surgical removal of these cancers, particularly in elderly people and those with other health conditions such as heart failure may not be necessary. Patients in Tayside Urological Cancers (TUCAN) database were carefully assessed using CT scans and discussed in multidisciplinary meetings and were followed up for more than 6 years. This kind of population-based study adds new knowledge to the understanding behaviour of a subset kidney cancers which otherwise have very poor outcome. The researchers and paper highlight careful documentation of cohort to understand natural history of disease.

**Abstract:**

There is emerging evidence to suggest that con-current medical conditions influence the outcome of cancers, irrespective of therapy offered. The prevalence and impact of co-morbidities on the survival outcome of complex renal cystic masses in not known. The objective was to study complex renal cysts (Bosniak ≥IIF ) and assess the overall and renal cancer-specific survival in a population-based database including impact of con-current morbidities. The Tayside Urological Cancer Network (TUCAN) database covering a stable population of more than 416,090 inhabitants in a defined geographical area identified 452 complex renal cysts in 415 patients between 2009 and 2019. Each patient was tracked and followed up using a unique identifier and deterministic linkage methodology. The last date of follow-up including cause of death was determined. Co-morbidities were recorded from primary care referrals. Renal cancer-specific mortality was 1.7% at a median follow-up of 76.0 months; however, overall survival was poor, particularly in patients ≥ 70 years of age and with ≥ 2 significant co-morbid conditions (*p* < 0.0001). A total of 38.3% of the cohort showed con-current morbidities. Age and co-morbidities were significant risk factors for overall survival in patients with complex renal cystic disease and a careful assessment should be made to recommend surgical intervention in the elderly population, in particular in those with other health-related conditions.

## 1. Introduction

The Bosniak classification stratifies renal cysts using radiological appearances to determine malignancy risk and guide management. Radiological features indicative of malignancy include irregular septa, nodular changes, wall thickening and significant enhancement [1,2].

Grounded by malignancy rates, the literature supports discharge of Bosniak I and II cysts, imaging follow-up of Bosniak IIF cysts and surgical intervention for Bosniak III and IV cysts. Interval imaging follow-up of Bosniak IIF cysts is necessary, because of the risk of progression and malignancy [3,4,5]. The likelihood and time to progression is undetermined in the literature. The progression rate of IIF in our selected population has been reported as 4.6%, with all malignant cysts progressing within 16 months) [6]. Malignancy rates of surgically resected IIF Bosniak cysts in our population is 60.0% (Lucocq, J) [6]. Other studies report a low progression rate but a high malignancy rate of resected progressed Bosniak IIF cysts [3,7]. A minimum of a 1 year follow-up for uncomplicated and up to 4 years for more complex Bosniak IIF cysts has been suggested [1,8].

The malignancy risk of Bosniak III cysts is variable in the literature. The evidence reports a malignancy rate between 33–84%; thus, benign cysts are frequently resected [8,9,10,11]. When malignant on histopathology, Bosniak III cysts are frequently low ISUP grade and stage [5,12,13]. In our population cohort, the malignancy rate of resected Bosniak III cysts is 79.3% [6]. Of all the resected cysts (Bosniak ≥ IIF), 73.8% are low ISUP (International Society of Urological Pathology) stage and 93.7% are confined to the kidney [6]. That raises questions regarding the careful selection of these patients for surgical interventions and associated survival benefits, especially in those with significant con-current health related conditions.

The long-term survival of patients with Bosniak III and IV cysts is unknown within the literature and limited to a few studies [5,14]. More specifically, the survival of cysts treated surgically versus managed conservatively has never been compared and warrants investigation, especially in those with co-morbid conditions. The identification of a cystic renal mass on imaging and the fear of missing a treatable mass often dominates care of these patients without considering survival impact of comorbidities.

Therefore, the objectives of this study were:

- To analyse the long-term overall and renal cancer-specific survival outcome of patients with complex cysts, and to compare survival between surgically versus conservatively managed cysts;
- To examine the distribution of comorbidity in this patient cohort and its impact on survival outcome.

## 2. Results

### 2.1. Overview of Complex Cysts

There were 317 cysts classified as ≥IIF using the Bosniak classification in 297 patients. The average patient age was 65.9 years (range, 20.5–93.8) and there were 198 males (66.7%). There were 161 category IIF cysts (149 patients), 79 category III cysts (71 patients) and 77 category IV cysts (77 patients). Fourteen percent of patients (42/297) required imaging with MRI either during characterization or throughout surveillance. The details of the resected cysts in this cohort have previously been described in a previous paper [6]. Briefly, the malignancy rates of IIF, III and IV cysts were 60% (3/5), 79.3% (23/29) and 84.8% (39/46), respectively. Of all malignant tumours, 73.8% were of low ISUP grade, 93.7% of tumours were confined to the kidney (stage pT2b or less) and 81.0% were less than 7 cm (stage pT1a or pT1b). Of the resected Bosniak III and IV cysts, 89.5% and 69.2% were of low ISUP grade, respectively. The recurrence rate for all resected cysts was 1.5% (1/65) after a median follow-up time of 50.2 months. A slight majority of cysts (53%) were resected with a partial nephrectomy versus a total nephrectomy [6].

### 2.2. Survival

Among the 292 patients followed up over a median observation period of 76.0 months (range, 0.1–175.5 months) there were 79 deaths: 69 deaths from a cause unrelated to renal cancer and five deaths from renal cancer. The mean survival time of patients who died was 22.8 months. Among those who died due to renal cancer, two had Bosniak IIF cysts, one had a Bosniak III cyst and two had Bosniak IV cysts at the time of diagnosis.

#### 2.2.1. Renal Cancer-Specific Survival—Surgical Versus Conservative Management in Bosniak III and IV Cysts

**Figure 1:**
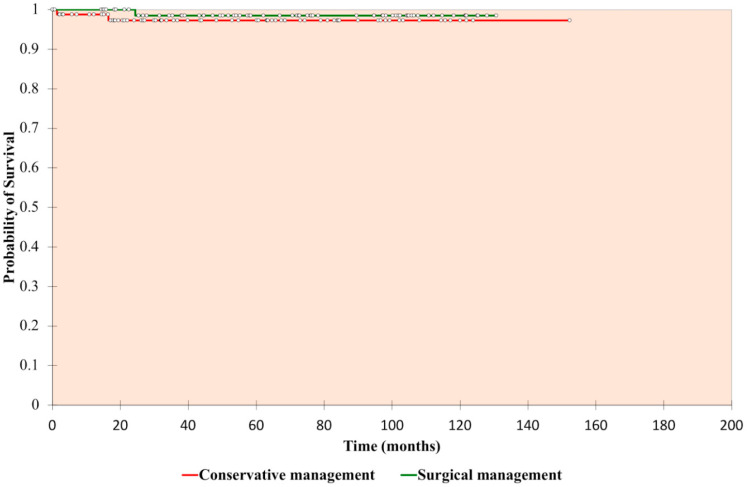
Kaplan-Meier analysis—renal cancer-specific survival of Bosniak III and IV cysts, surgical versus conservative management.

The death rate from renal cancer for all complex cysts was 1.7% (5/292). The renal cancer-specific mortality rate in the surgical and conservatively-managed groups was 1.2% (1/81 cysts; median follow-up, 77.6 months) and 2.7% (2/75 cysts; median follow-up, 77.6 months), respectively (*p* = 0.54, Figure 1, Appendix B). The median age of the group treated surgically versus the group treated conservatively was 63 years versus 75 years (*p* < 0.0001). A total of 2.5% of patients in the surgical group had two or more significant comorbidities, versus 29.3% in the conservative group. The 10-year probability of renal cancer-specific survival for surgical management versus conservative management was 0.985 and 0.973, respectively. Once poor surgical candidates were excluded, there was also no significance between groups (*p* = 0.24).

#### 2.2.2. Overall Survival—Associations with Unrelated Cause of Death

**Figure 2:**
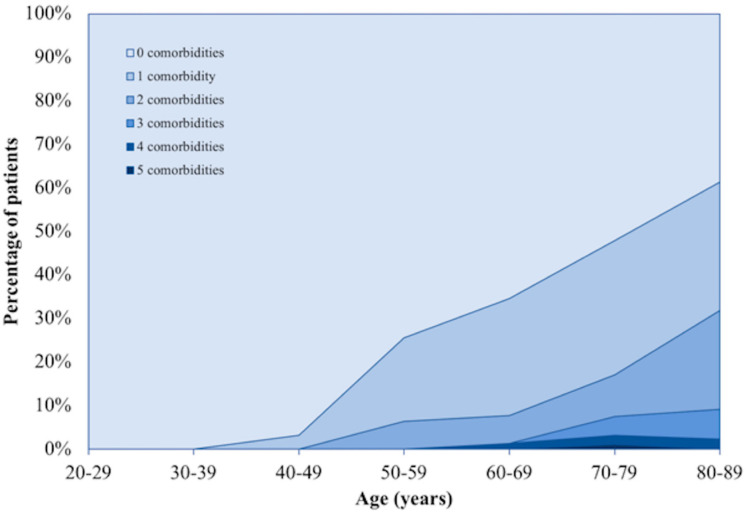
The number of significant comorbidities by age-group.

**Figure 3:**
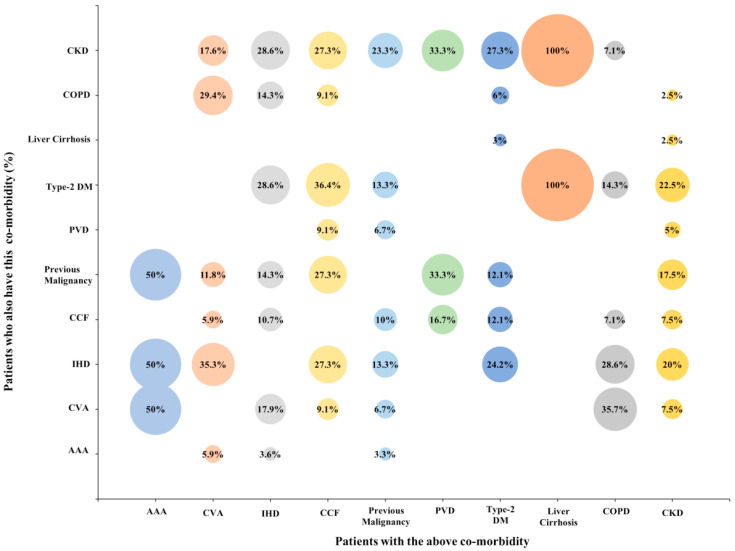
The percentage of patients with one significant comorbidity who have another significant comorbidity.

The burden of significant comorbidities is high in patients with complex renal cysts, particularly in the elderly population (Figure 2 and Figure 3). At the time of diagnosis, the Charlson Comorbidity Index (CCI) for those who died versus survived was 70.6% compared to 30.8%, (*p* < 0.00001). The mean age of the two groups was 73.8 years and 64.7 years, respectively (*p* < 0.00001).

For complex cysts ≥ IIF, ≥2 significant comorbidities (HR—3.296; *p* < 0.0001; 95% CI—1.914–5.675) and age ≥ 70 years (HR—4.945; *p* < 0.0001; 95% CI—2.672–9.153) were associated with a higher risk of death (Appendix C). In the model with Bosniak III and IV cysts alone, the presence of ≥2 significant comorbidities (HR—2.890; *p* = 0.002; 95% CI—1.470–5.681) and age ≥ 70 years (HR—5.020; *p* < 0.0001; 95% CI—2.350–10.721) were associated with a higher risk of unrelated death (Appendix C). In this model, being female was associated with better survival (HR—0.438; *p* = 0.017; 95% CI—0.222–0.864). A separate Cox proportional hazard model incorporated the significant comorbidities as separate variables, in which ischaemic heart disease, peripheral vascular disease, previous malignancy, chronic kidney disease, chronic obstructive pulmonary disease, age and Bosniak IV category, were associated with death (*p* < 0.05, Appendix C). Of all the significant comorbidities, patients with Bosniak IV cysts were more likely to have peripheral vascular disease (*p* = 0.005) and chronic kidney disease (*p* = 0.01).

**Figure 4:**
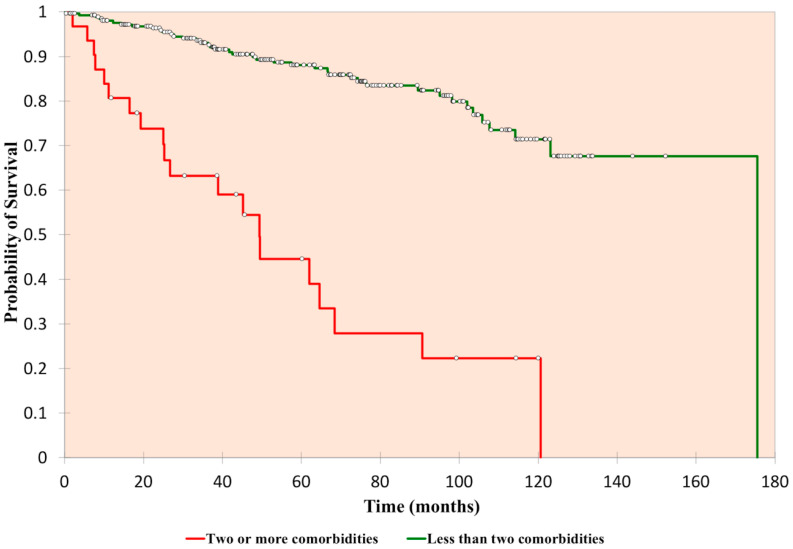
Kaplan-Meier analysis—overall survival of all complex cysts, two or more comorbidities versus less than two comorbidities.

The probability of five-year survival within the group of patients with ≥2 significant comorbidities versus <2 comorbidities was 0.45 and 0.88, respectively (*p* < 0.0001, Figure 4, Appendix B). The same test was conducted for Bosniak III and IV, and the 5-year probability of survival was 0.47 versus 0.83, respectively (*p* < 0.0001) (Appendix B). In those who were less than 70 years of age and with less than two co-morbidities, the probability of five-year survival was 0.92. In this sub-cohort of 168 patients, there were 15 deaths (8.9%) over a median follow-up period of 68 months.

## 3. Discussion

The unnecessary resection of benign Bosniak III cysts is a recognised limitation of the Bosniak classification. The addition of the intermediate IIF category has improved the clinical significance of the Bosniak classification, as evidenced by an increased malignancy rate of surgically resected Bosniak III cysts and decreased rate of surgery. Since the incorporation of IIF cysts into the classification system, studies have reported malignancy rates for Bosniak III cysts of 60% and 81.8% [8,10,15,16]. A meta-analysis by Graumann et al. found that the malignancy rate of Bosniak III was 65.4% [17]. In our cohort, the malignancy rate of Bosniak III cysts is 79.3% and the rate of benign cyst resection is relatively low. Nevertheless, resection of benign cysts persists as a significant proportion of resections and patients frequently undergo unnecessary surgery. Furthermore, Bosniak IV cysts can be benign, despite exhibiting radiological features indicative of malignancy, and there is also a risk of benign cyst resection in these patients [11].

The ISUP grading system is employed to predict the biological aggressiveness of the cancer. An ISUP grade of 1 or 2 predicts a relatively indolent cancer. Cystic clear cell renal cell carcinomas are generally low grade, irrespective of Bosniak category [12,13]. A period of observation may help select progressing cysts, increase the malignancy rate of resected Bosniak III cysts and reduce the proportion of benign cyst resections. Further studies are warranted to investigate if a period of observation would reduce the rate of surgery, whilst not compromising outcome.

The literature on the survival of patients with Bosniak cysts is limited to a few studies [5,14,18]. In this study, the overall survival of all complex cysts was low and was secondary to higher age and a high prevalence of significant comorbidities. Elderly patients (>70 years of age) with co-morbid conditions were more likely to have mortality from non-cancer specific causes than due to renal cancer. In these patients, the renal cancer specific survival is very high, and there was no significant difference in survival between groups managed surgically versus conservatively. These findings in combination with the low grade and stage of cancers suggests that Bosniak III and IV cysts, particularly Bosniak III cysts, should be managed conservatively in the older patient and those with significant co-morbid conditions. This is a significant finding of the study and should prompt decision-makers to plan interventions including surgical resection on an individualised basis according to age and comorbidities [14]. Chandrasekar et al. reported an overall mortality of 6.2% in contrast to our study, where our observed non-cancer specific death rate was 23.2% (69/292) overall, and 8.9% (15/168) in those less than 70 years of age, and with less than two significant comorbidities. Both the mean age of their patient cohort and the median follow-up time was lower than that in our study and follow-up was shorter in comparison. They did not analyse detailed impact of co-morbid conditions on the survival outcomes, but it is likely that we had patients with a higher number of co-morbid conditions. Consistent with our findings, they report a low mortality from renal cancer (0.3%) [14].

It is well-established that comorbidities and poor general health have significant impact on the outcome of individual conditions. Still, most guidelines are formulated for conditions without recommendations for assessing multimorbidity and hence may not be applicable to representative population from a defined geographical area with varying socioeconomic index [19,20]. Clinicians recommending treatment options based on disease-specific guidelines without taking into account measurement of comorbidities may put these patients at risk of potentially harmful adverse events without improvement in survival or treatment-specific advantages [21]. Adverse events witnessed in this study were the resection of benign Bosniak III and IV cysts, surgical complications and long-term radiation exposure. In the present study, the survival advantage of surgical intervention was not significant, particularly in elderly patients (>70 years) and those with two or more than two co-morbid conditions. Findings of the present study should generate debate around frequently subjecting these patients to contrast enhanced CT scans, the benefit of surgical intervention and associated management issues including management of co-morbidities and complications. Instead of discharging patients entirely from further follow-up, surveillance interval ultrasound scans would be indicated to monitor progression of the cysts.

Future studies are recommended to establish the burden of comorbidities in patients with complex renal cysts across other geographical regions. The prevalence of significant co-morbidities in this study was high (38.3%) and, as a result, the overall survival was low. A high burden of comorbidities and low overall survival across multiple studies would indicate that current management needs to be revised and a more conservative approach adopted. Conversely, it is possible that the high rate of co-morbidities is not specific to patients with complex renal cysts and that proposed management should differ dependent on geographical area and socioeconomic index.

The main strength of this study is that the cohort could be observed throughout the 11-year period, and we could gain live information on survival in all the patients using data linkage methodology. The study had limitations, as there were a small number of cases where the cause of death was uncertain and, hence, excluded from the final analysis; however, that should not change the major findings and their potential impact in the future.

## 4. Methods

### 4.1. Definition of Population Cohort

**Figure 5:**
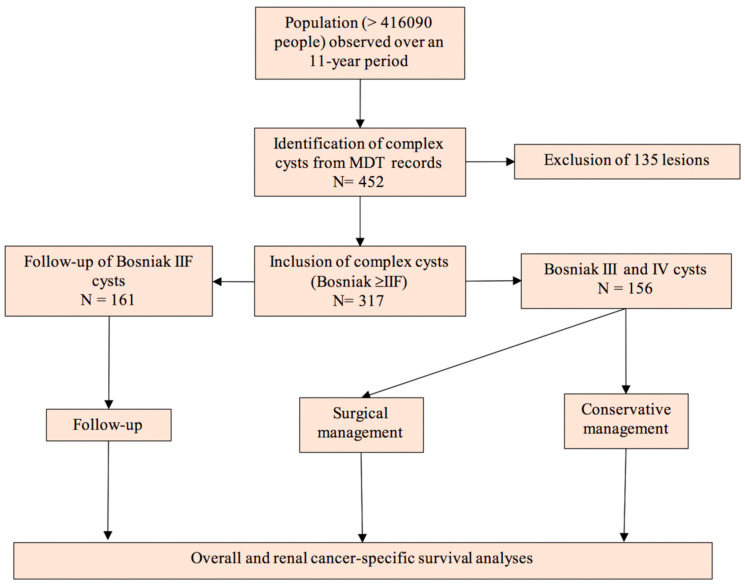
Study design.

The Tayside Urological Cancer Network (TUCAN) database stores the health records of patients with complex renal cysts diagnosed in a defined geographical area with a population of more than 416,090 people based on mid-year 2017 population estimates [22]. All patients with renal cysts diagnosed between January 2009 and December 2019 were recorded in the TUCAN database and reviewed at multi-disciplinary meetings by an uroradiologist. The discussions were recorded on a proforma (Appendix A) and patients were followed-up with interval imaging as necessary and tracked using a unique Community Health Index (CHI) number. For the purposes of investigating complex cysts, Bosniak I and II cysts, haemorrhagic cysts and inflammatory cysts were excluded from analysis. Haemorrhagic cysts were diagnosed by haemorrhagic material within the cyst and the absence of Bosniak ≥ IIF criteria. Figure 5 illustrates the study design.

### 4.2. Data Collection

The data from each patient with renal cysts was documented on a secure electronic database and linked to other electronic databases using the CHI number. The deterministic records-linkage methodology between systems provided radiological data, details of surgical intervention and the data of the resected specimen such as histologic subtype, grade and stage. Overall and renal cancer-specific survival for each patient was accessible through the electronic databases. The validity of linkage method was high as besides unique number, we used date of birth, sex and post code of patient.

Multimorbidity data was identified from the referral letters to the multi-disciplinary meeting. The ten most prevalent comorbidities constituting the Charlson Comorbidity Index were considered in analysis and compared within groups to evaluate who would benefit from surgical intervention [23].

### 4.3. Follow-up Protocol

Patients with Bosniak IIF cysts were followed-up in accordance with the local protocol with serial imaging at 6, 12, 24, 36 and 48 months. In cases of high likelihood of contrast nephropathy, MRI with gadolinium contrast imaging was used. If the follow-up imaging demonstrated an increased complexity of the cyst or a significant increase in size, the case was referred to the multi-disciplinary meeting and the imaging was reviewed by the uroradiologist. Progression of cyst complexity was defined by increased cyst complexity according to the Bosniak criteria. Radiological findings attributable to cyst progression include progressing enhancing nodules, septal thickening and wall thickening [2]. Patients with Bosniak III and IV cysts underwent surgical intervention with either a partial or total nephrectomy subject to patient and cyst-specific factors. Those not managed surgically were either discharged from follow-up or were followed-up with interval imaging.

### 4.4. Imaging Protocol

**Figure 6:**
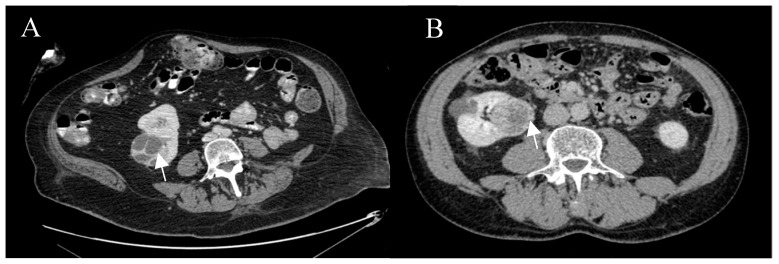
Post-contrast axial CT images of complex right renal cysts. (**A**) Bosniak IV cyst, conventional clear cell, pt3a, grade 2, resected by radical nephrectomy. (**B**) Bosniak IV cyst, oncocytoma, resected by partial nephrectomy.

There is an established protocol to characterise cysts at our institution as described before [5]. The CT renal characterisation scan is triphasic with precontrast, arterial (40 s post-contrast) and nephrographic phases (100 s post-contrast), following IV administration of 100 mL of iodine-based contrast agent (omnipaque) and acquired as a volume using a 64-slice thickness CT scanner (Figure 6). The 100 s delay helps to visualise the kidneys in their nephrographic phase; the optimal phase to characterise renal cysts. The nephrographic phase was also obtained using MRI and gadolinium contrast. T2 HASTE (6 mm-slice thickness, 1.8 mm gap), T1 VIBE pre- and post-contrast (3.5 mm-slice thickness, 0.7 mm gap), STIR (8 mm-slice thickness, 2.4 mm gap) and diffusion-weighted (4 mm-slice thickness, 1.2 mm gap; b-values 50, 400, 1000 s/mm^2^) images were obtained. The change in cyst enhancement was measured by calculating the difference in pre- and post-contrast enhancement.

### 4.5. Survival Analyses

The overall and renal cancer-specific survival of Bosniak cysts ≥ IIF was calculated using Kaplan-Meier curves. Survival was calculated as the time from diagnosis to death or until the end of the data collection period (in these cases the patients were censored). The renal cancer-specific survival was compared between surgical management and conservative management groups and Log-Rank and Wilcoxon tests were used to compare survival curves. Within the overall survival curves, cox proportional hazards models interpreted the hazard ratio of comorbidities, age and sex. The Charlson Comorbidity Index (CCI), a validated model for estimating the 10-year mortality was calculated and the mean CCI of groups were compared using the Student’s t-test. Patients who died of unknown causes were excluded from the survival analysis.

## 5. Conclusions

The renal cancer-specific survival is very high and most deaths in complex renal cystic disease are related to non-cancer specific causes. Age and comorbidities are significant risk factors for overall survival in patients with complex renal cystic disease, and a careful assessment should be made to recommend surgical intervention in the elderly population, in particular for those with other health-related conditions. In those who are not treated with surgical management after consideration of age and comorbidities, interval ultrasound scans can be performed to monitor progression of the cysts, while limiting radiation exposure.

## Figures and Tables

**Figure A1:**
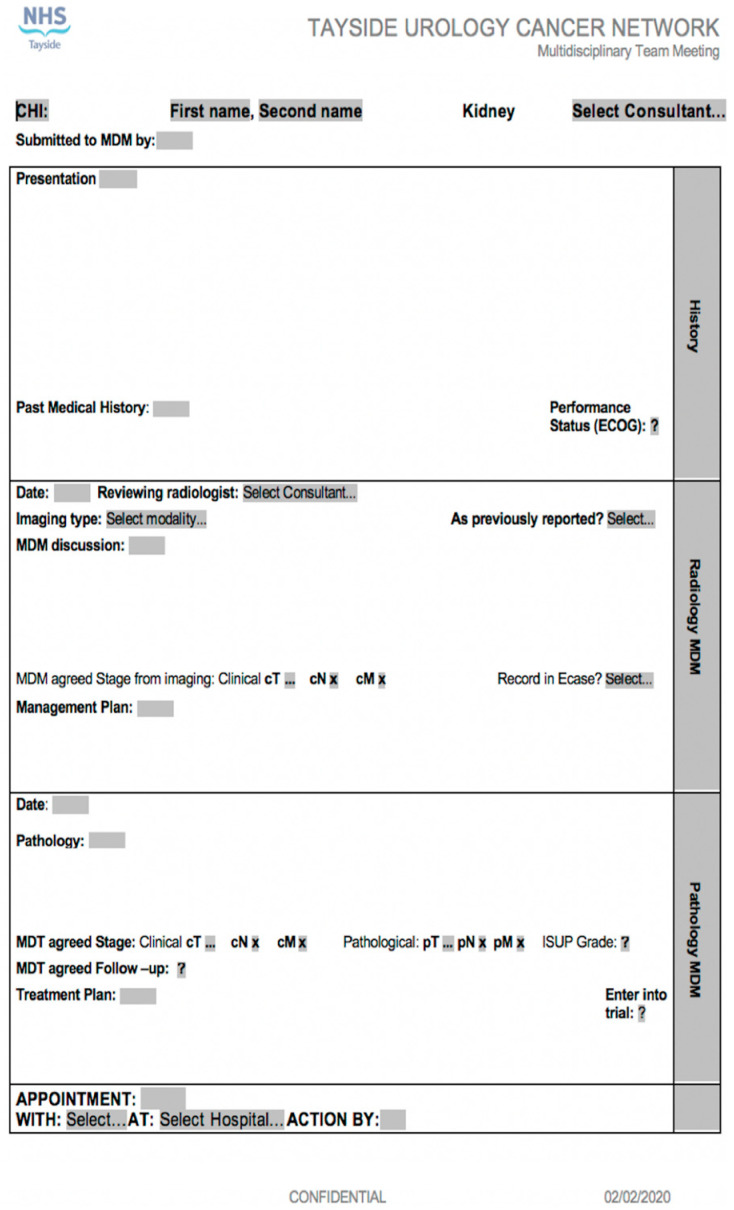
Proforma.

## Appendix B

**Table A1:** Kaplan-Meier analysis, comparison of survival.

Statistic	Observed Value	Critical Value	*p*-Value	alpha
Surgical versus conservative management (renal cancer-specific survival)				
Log-rank	0.367	3.841	0.544	0.050
Wilcoxon	0.524	3.841	0.469	0.050
≥2 Comorbidities versus <2 comorbidities (overall survival)				
Log-rank	9.226	5.991	0.010	0.050
Wilcoxon	8.058	5.991	0.018	0.050
≥2 Comorbidities versus <2 comorbidities (Bosniak III and IV cysts; overall survival)				
Log-rank	9.226	5.991	0.010	0.050
Wilcoxon	8.058	5.991	0.018	0.050

## Appendix C

**Table A2:** Cox proportional hazards model—associations between variables and death (Bosniak ≥ IIF cysts).

Variable	Value	Standard Error	Wald Chi-Square	*p*-Value	Hazard Ratio	Hazard Ratio Lower Bound (95%)	Hazard Ratio Upper Bound (95%)
Bosniak ≥ IIF cysts							
Age (≥ 70 years)	1.598	0.314	25.890	<0.0001	4.945	2.672	9.153
Female	−0.469	0.283	2.747	0.097	0.691	0.359	1.090
Bosniak III	0.241	0.318	0.575	0.448	1.206	0.683	2.371
Bosniak IV	0.947	0.297	10.184	0.001	2.578	1.441	4.613
Two or more comorbidities	1.193	0.277	18.501	<0.0001	3.296	1.914	5.675
Bosniak III and IV cysts							
Age (≥70 years)	1.613	0.387	17.368	<0.0001	5.020	2.350	10.721
Female	−0.827	0.347	5.670	0.017	0.438	0.222	0.864
Bosniak IV	0.733	0.320	5.253	0.022	2.082	1.112	3.899
Two or more comorbidities	1.061	0.345	9.468	0.002	2.890	1.470	5.681
Bosniak ≥ IIF cysts (comorbidities as single variables)							
Age	0.063	0.017	14.081	0.000	1.065	1.031	1.101
Female	−0.391	0.320	1.499	0.221	0.676	0.361	1.265
Bosniak III	0.001	0.367	0.000	0.997	1.001	0.488	2.055
Bosniak IV	0.720	0.338	4.545	0.033	2.055	1.060	3.984
Abdominal Aortic Aneurysm	0.867	1.071	0.656	0.418	2.380	0.292	19.411
Cerebrovascular disease	0.492	0.497	0.982	0.322	1.635	0.618	4.328
Ischaemic Hear Disease	0.963	0.365	6.941	0.008	2.619	1.280	5.360
Heart Failure	0.868	0.490	3.131	0.077	2.381	0.911	6.227
Previous Malignancy	0.750	0.346	4.694	0.030	2.117	1.074	4.172
Peripheral Vascular Disease	1.283	0.606	4.479	0.034	3.607	1.099	11.832
Type-2 Diabetes Mellitus	0.075	0.465	0.026	0.872	1.078	0.433	2.681
Liver Cirrhosis	−15.363	1761.730	0.000	0.993	0.00	0.000	-
Chronic Obstructive Pulmonary Disease	1.007	0.508	3.931	0.047	2.736	1.012	7.402
Chronic Kidney Disease	0.889	0.311	8.154	0.004	2.432	1.321	4.476
